# Immunotherapy targeting toxic Tau reduces and mitigates cognitive decline, and senescence in a human Tau mouse model

**DOI:** 10.1002/alz.089755

**Published:** 2025-01-03

**Authors:** Fadhl Al‐Shaebi, Sudipta Senapati, Alessia Sciortino, Marina Farag, Madison Samples, Rhea Xavier, Rakez Kayed

**Affiliations:** ^1^ University of Texas Medical Branch, Galveston, TX USA

## Abstract

**Background:**

Alzheimer’s disease (AD) is characterized by the accumulation of tau protein in the brain, which forms neurofibrillary tangles and contributes to the gradual deterioration of brain function. As a consequence, cellular senescence occurs, leading to cognitive impairment and hastening the aging process. Immunotherapies targeting Aβ and other protein aggregates are also being developed in the meantime. This study looks at immunotherapy in a Mapt (hTau) mouse model. This could become a useful immunological treatment in the future by lowering inflammation and cellular senescence that come with getting older.

**Method:**

Mapt (hTau) mice were inoculated in the hippocampus with 1 µg of brain‐derived Tau oligomers (BDTOs) from AD patients. After seven months, mice were injected in the tail vein with 120 µg of in‐house mouse monoclonal anti‐toxic tau antibodies (TTCM1‐2 and TOMA1‐4) or IgG isotype control. Mice were evaluated for cognitive and motor function before euthanasia, and brain neuropathology was investigated with immunohistochemistry, immunofluorescence, and western blot techniques.

**Result:**

Treatment with anti‐toxic tau antibodies reduced total tau levels and tau phosphorylation in the brain. Furthermore, immunotherapy reduced inflammation and cellular senescence markers.

**Conclusion:**

The observed decrease in tau aggregates following immunotherapy indicates that the mechanisms responsible for tau phosphorylation and/or aggregation may have been altered. Additionally, the decrease in senescence and inflammation markers suggests that immunotherapeutic strategies targeting tau are a promising approach in the management of tauopathies.

